# Estimating program coverage in the treatment of acute malnutrition using population-based cluster survey methods: results from surveys in Burkina Faso, Chad, Democratic Republic of the Congo, and Niger

**DOI:** 10.3389/fpubh.2025.1513567

**Published:** 2025-03-25

**Authors:** Grace Heymsfield, Elizabeth Radin, Marie Biotteau, Suvi Kangas, Zachary Tausanovitch, Casie Tesfai, Léonard Kiema, Wenldasida Thomas Ouedraogo, Badou Seni Mamoudou, Mahamat Garba Issa, Lievin Bangali, Marie Christine Atende Wa Ngboloko, Balki Chaïbou, Maman Bachirou Maman, Eva Leidman, Oleg Bilukha

**Affiliations:** ^1^International Rescue Committee, New York, NY, United States; ^2^International Rescue Committee, Dakar, Senegal; ^3^International Rescue Committee, Brussels, Belgium; ^4^International Rescue Committee, Ouagadougou, Burkina Faso; ^5^Ministère de la Santé et de l'Hygiène Publique, Ouagadougou, Burkina Faso; ^6^International Rescue Committee, N'Djamena, Chad; ^7^Ministère de la Santé Publique et de la Prévention, N'Djamena, Chad; ^8^International Rescue Committee, Goma, Democratic Republic of Congo; ^9^Programme National de Nutrition (PRONANUT), Ministère de la Santé Publique, Hygiène et Prévention Secrétariat Général, Kinshasa, Democratic Republic of Congo; ^10^International Rescue Committee, Niamey, Niger; ^11^Ministère de la Santé Publique, de la Population et des Affaires Sociales, Niamey, Niger; ^12^US Centers for Disease Control and Prevention, Atlanta, GA, United States

**Keywords:** cluster survey, coverage, SQUEAC, severe acute malnutrition, therapeutic feeding program

## Abstract

**Background:**

Despite their utility for program planning, acute malnutrition treatment coverage estimates at the national and sub-national levels are rarely available. Prior work has identified methodological concerns with current approaches.

**Methods:**

We estimated the point prevalence and treatment coverage of acute malnutrition in 11 districts (or similar subnational areas) across four high-burden countries in Africa using representative cluster-based population survey methods and compared these estimates to those derived from administrative data and other direct methods where available. We also aimed to assess information about risk factors for malnourished children by coverage status.

**Results:**

The point estimate of coverage suggests that <20% of eligible children with severe acute malnutrition (SAM) were enrolled in treatment in nine administrative areas. We found that in some contexts, coverage estimates derived using administrative data are useful, while in others, they are not – and that their accuracy can vary by month and year. By comparison, coverage estimates from other direct methods were overestimated and/or outdated, and practitioners tended to overestimate coverage. Coverage did not differ significantly by sex or age of the child but did vary by mid-upper arm circumference (MUAC) at assessment. Measured SAM coverage did not correlate either with measured SAM prevalence or with expected coverage estimated *a priori* by program staff.

**Conclusion:**

Our findings suggest that in the assessed high-burden countries, many more children are eligible for treatment than are enrolled. We present this methodology as an alternative to existing primary methods and a complement to coverage estimates from routine program and population data.

## 1 Introduction

Acute malnutrition, or wasting, is a biological coping mechanism to disease, infections, or inadequate dietary intake characterized by a rapid deterioration in nutritional status. An estimated 45 million children suffer from acute malnutrition (1). Analysis of historical cohort data suggests that severe acute malnutrition (SAM) is associated with an 11-fold increase in the risk of death, while moderate acute malnutrition (MAM) is associated with a 3-fold increase compared to children without acute malnutrition living in the same communities (2).

Routine treatment of severe acute malnutrition among children without medical complications includes the use of ready-to-use therapeutic food (RUTF), usually in the form of a fortified peanut paste, as part of community-based management of acute malnutrition (CMAM) programs (3, 4). Evidence from select programs with strong programmatic support and supervision suggests a high proportion of children recover from acute malnutrition when treated using CMAM protocols implemented with fidelity (3, 5, 6). Unpublished routine data suggests International Rescue Committee (IRC) programs meet the SPHERE standards for the management of severe acute malnutrition, with over 75% recovery rates (7).

While effective, these programs face various supply and demand barriers to scale, including underfunding, frequent stockouts of supply, health systems constraints, sociocultural barriers and insecurity (8, 9). Therefore, ensuring all children with acute malnutrition are able to access care remains a challenge globally. Geographic coverage (often measured as the percent of all health facilities offering services) is typically higher than treatment coverage (percent of eligible children receiving care) (8–10). As a result, despite relatively straightforward treatment protocols (compared, for example, to antiretroviral therapy for HIV/AIDS) and being highly effective, modeled estimates of coverage of wasting treatment suggest coverage remains low, but these estimates are sparse. Based on the total number of children with SAM treated and the total estimated SAM burden, estimated treatment coverage changed from 33%in 2018 to 39.8% in 2019 (11). We were not able to identify a similar global estimate for MAM.

Despite their utility for program planning, management, and monitoring purposes, treatment coverage estimates at the national and sub-national levels are rarely available (12). While coverage of other interventions such as vaccines are regularly integrated in large-scale surveys (Multiple Indicators Cluster Survey (MICS), Demographic and Health Survey (DHS)) using probability sampling methods and substantial technical assistance, the sample size of severely malnourished children is often too small to ascertain program coverage of SAM and MAM (13–15). Thus acute malnutrition treatment coverage has typically been calculated using one of three approaches: (1) “indirect” estimates using administrative data, (2) “direct” estimates using either Semi-Quantitative Evaluation of Access and Coverage (SQUEAC), or (3) Simplified Lot Quality Assurance Sampling Evaluation of Access and Coverage (SLEAC) (16, 17). Prior work has identified methodological concerns with each of these three approaches (16, 18, 19).Estimates using administrative data are derived by dividing reported admissions by the estimated eligible population. While this approach requires the fewest assumptions, the accuracy of the calculation depends on the availability and accuracy of administrative data, including admission figures, population figures, prevalence estimates, and the average duration of a malnutrition episode (16). Given these challenges, coverage can also be assessed through cross-sectional assessments designed specifically for measuring coverage. SQUEAC and SLEAC are methodologies for such assessments. The SQUEAC method is a conjugate Bayesian analysis of a formulated expected coverage (‘prior’) and small-scale survey using active and adaptive case finding (AACF), which uses case definitions to snowball sample children (17). The final coverage estimate can be biased by the formulated prior (18), and AACF has been demonstrated to introduce upward bias (19). Furthermore, given that acute malnutrition is a sensitive and stigmatized condition across a wide range of contexts (9, 20–22), it is worth considering whether this form of case finding could cause social harm (19). The SLEAC is a similar method used to classify and map coverage, typically at a larger geographic level of aggregation. In contrast to a SQUEAC, SLEAC provides limited information on barriers and does not produce a coverage estimate with an associated confidence interval, but uses the same AACF approach during primary quantitative data collection (17).

The cluster survey method for estimating the coverage of acute malnutrition presented here is proposed as an alternative methodology that aims to limit the biases present in the other methodologies. The proposed approach limits the possibility of selection bias through an exhaustive enumeration of households within selected clusters. While the approach involves more time intensive primary data collection, it aims to provide more reliable measures of treatment coverage and allows for concurrent estimation of prevalence. The approach has been conducted in prior research settings, but never at scale for programmatic benefit (18).

To address the lack of reliable coverage estimates at the subnational level, we estimated the point prevalence and treatment coverage of acute malnutrition in 11 districts (or similar subnational areas) across four high burden countries in Africa using representative cluster-based population survey methods.

Our research question was: what is the program coverage of acute malnutrition treatment when measured using population-based cluster surveys, and can program coverage measured using these methods be compared to indirect estimates using administrative data or other direct estimates? We report our findings from eleven distinct administrative areas in four countries to demonstrate relevance to other contexts where acute malnutrition treatment programs are offered. We compared these estimates to those derived from administrative data and other direct methods where available. We also assessed information about risk factors for malnourished children by coverage status.

## 2 Methods

### 2.1 Survey settings

Eleven surveys were conducted in the catchment areas of health facilities supported by the IRC CMAM Avancé project for the treatment of SAM in Burkina Faso, Chad, Democratic Republic of the Congo (DRC), and Niger between September 2021 and January 2022 (Table 1).

**Table 1:** Description of survey settings in terms of treatment services in place and catchment population served.

	Burkina Faso			Chad			DRC		Niger		
	Health district			Health district			CMAM Avancé catchment areas within health zones		Health district		
	Bogodogo	Boulmiougou	Sig-Noghin	Baro	Mangalme	Melfi	Kalemie	Nyemba	Balleyara	Filingué	Ouallam
Context	Urban	Urban/Peri-urban	Urban/Peri-urban	Rural	Rural	Rural	Rural/Peri-urban	Rural/Peri-urban	Rural	Rural	Rural
SAM Protocol	WHZ < −3, MUAC <115 mm, and/or edema			MUAC <115 mm, and/or edema			WHZ < −3, MUAC <115 mm, and/or edema		WHZ < −3, MUAC <115 mm, and/or edema		
MAM Protocol	WHZ ≥ −3 and WHZ < -2, MUAC ≥115 mm– < 125 mm			MUAC ≥115 mm to <125 mm			WHZ ≥ −3 and WHZ < −2, MUAC ≥115 mm– < 125 mm		WHZ ≥ −3 and WHZ < −2, MUAC ≥115 mm– < 125 mm		
Continuity of treatment	Treatment supported by MoH			Treatment supported by MoH. Stockouts June – August 2021.			Inconsistent treatment due to lack of supplies		Treatment supported by MoH		
Total facilities											
# static OTP facilities	40	47	29	9	12	11	17	2	11	18	21
# static TSFP facilities	40	47	29	9	12	11	15	2	11	18	21
# mobile sites (SAM + MAM)	0			0			0		0		
Population 6–59 months	122,674	131,236	49,619	13,552	28,445	22,589	48,071	16,075	23,347	72,552	51,402
Children 6–59 mo: Facility	3,067	2,792	1,711	1,506	2,370	2,054	2,828	8,038	2,122	4,031	2,448
Stabilization Centers	1	1	0	1	1	1	2	1	1	1	1
Screening activities											
National vaccination campaigns	3x annually			3x annually			No		3x annually		
Active screening	Irregularly implemented by CHW’s^2^			Irregularly implemented by CHW’s			Inactive.		Irregularly implemented by CHV’s.		
Passive screening at the facility	Yes			Yes			Yes		Yes		
Family MUAC	No			Yes	Yes	Yes	No		Yes	Yes	Yes
Survey dates	September 28 – November 1, 2021			October 13–November 6, 2021			November 12–December 9, 2021		December 24, 2021- January 14, 2022		

MAM, Moderate Acute Malnutrition; MoH, Ministry of Health; MUAC, mid-upper arm circumference; OTP, Outpatient Therapeutic Program; SAM, Severe Acute Malnutrition; TSFP, Targeted Supplementary Feeding Program; WHZ, Weight-for-Height z-score.

^1As per the administrative data used in the most recently available SMART or other population-based survey in each context.^

^2^Community health workers (CHW’s) are community members who work either for pay or as volunteers (CHV’s) in association with the local health care system.

Strategies aimed at increasing coverage funded by the CMAM Avancé project, such as mass screening campaigns and mid-upper arm circumference (MUAC) screening by caregivers, did not start until after the coverage surveys. However, programs providing treatment for acute malnutrition were operational in most administrative areas for several years prior to when coverage surveys were conducted (Table 1). Notable disruptions in service provision in the months preceding the surveys are also noted in the table, including regional disruptions associated with RUTF stockouts in DRC in areas where partners were not supporting treatment and national stockouts in Chad (June – August 2021).

Additionally, active outreach activities were ongoing to identify children eligible for treatment and refer them for care in some surveyed settings. Mass screening was integrated into national vaccination campaigns in Burkina Faso, Chad, and Niger. Active screening was also implemented by compensated community health workers or uncompensated volunteers in all countries, except for DRC, where the community health volunteer network was inactive in the catchment areas. In addition to active outreach by trained providers in select countries, caregivers were provided with MUAC tapes and trained to use the tapes to monitor the nutritional status of their children in Chad and Niger (Family MUAC).

### 2.2 Sampling methodology

For each administrative area assessed, sampling was conducted in two stages: cluster sampling at the village or other primary sampling unit (PSU) level and exhaustive sampling of children within the clusters.

Target sample size, expressed in the number of clusters, was proportional to the average number of households per cluster and expected SAM prevalence (Equation 2) (18). The required sample size in terms of the number of children with severe acute malnutrition (SAM children) required in that formula was estimated using Equation 1 (18). The parameters for sample size calculations are included in Supplementary File 1.


(1) $$n\;SAM\;\mathrm{children}=\left(\frac{\begin{array}{l} \mathrm{expected}\;\mathrm{coverage}\;x\;\\ \left(1-\mathrm{expected}\;\mathrm{coverage}\right) \end{array}}{{\left(\frac{\mathrm{precision}}{1.96}\right)}^{2}}\right)$$

MAM coverage was not assessed in Burkina Faso or Chad, where supplies for referral facilities were not available at the time of data collection.

Expected coverage estimates were estimated by IRC technical staff in each country office, informed by stakeholder consultation, and a review of factors that can boost or inhibit coverage, including the food security situation, partner presence prior to the project, availability of MAM treatment, insecurity/ physical accessibility, population displacement, and available coverage survey data. Non-response was estimated to be 5% unless the rate in the most recent Standardized Monitoring and Assessment of Relief and Transitions (SMART) survey or Demographic and Health Survey (DHS) was higher. Desired precision ranged from 6.5–12%, based on the expected change in coverage after 1 year of implementation, as well as the operational feasibility. Expected prevalence of SAM and population age distribution were based on available data from the most recently available SMART or other population-based survey in each context. Average cluster size was calculated based on the list of all selected PSU’s (18).


(2) $$n\;\mathrm{clusters}=\left(\frac{n\;SAM\;\mathrm{children}}{\begin{array}{l} \mathrm{prevalence}\;SAM\;\mathrm{children}\\ {}*\mathrm{average}\;\mathrm{cluster}\;\mathrm{population}\\ {}*\%\mathrm{children}\;6-59\;\mathrm{months} \end{array}}\;\right)*\left(1+non-\mathrm{response}\;\mathrm{rate}\right)$$

The total number of households in the sample was higher in contexts where the expected SAM prevalence was lower. For full details and parameters used in sample size calculation, see Supplementary File 1.

Cluster selection was made using spatial stratified systematic sampling or probability proportional to size (PPS) depending on the context. Spatial stratified systematic sampling was conducted in contexts with less variation in cluster size, whereas PPS was preferred in areas with high variation in cluster size. In Burkina Faso, Niger, and DRC, for stratified systematic sampling, a complete list of clusters was sorted by geographically delimited health area, and villages were selected using systematic random sampling to yield a reasonably even spatial sample across health areas. In Chad, for probability proportional to size, selected clusters were selected using ENA (Emergency Nutrition Assessment) for SMART software (23). The list of PSU’s was based on the most recent population-based survey in each administrative area of the project.

### 2.3 Training and data collection

Enumerators received 4–5 days of training, including classroom-based training, a MUAC-based standardization test, and a pilot test. Data were collected by two-person teams, including an interviewer and a measurer. Enumerators were hired as interviewers if they achieved a Technical Error of Measurement (TEM) of 1.3 mm or better during the standardization exercise (24). Teams were supervised by IRC staff trained in nutrition, monitoring and evaluation, and the survey methodology.

All households in the selected cluster were administered informed consent. Consenting households were eligible for inclusion if they had a child less than 6 years old. Household definitions were based on the most recent SMART or other population-based survey. Household questionnaires were administered to the head of the household. Collected information included the number of children younger than 6 years old in the household, age, household distance to the health facility, and questions about care seeking. Age was recorded from birth certificates and vaccination cards where available, or estimated using local events calendar if not for all children younger than 6 years old. For children, 6–59 months of age, mid-upper-arm circumference (MUAC) and edema were assessed. Standard MUAC tapes were used for children 6–59 months. Bilateral pitting edema was assessed and verified by photo. All data were collected directly on tablets using the CommCare platform (Dimagi, Inc.) (25). Printed paper forms accompanied enumerators in case of issues with mobile data collection. All not currently treated cases were referred to the nearest treatment facility.

To improve data quality, ranges of values were programmed into CommCare for implausible data for select fields (i.e., MUAC <40 or > 250 mm). PowerBi dashboards were configured with CommCare for daily feedback to survey managers with an overview of areas assessed, enumerator performance, and flagged data. Additional analyses were conducted in RStudio by the headquarters Nutrition Specialist every other day during data collection. Quality control procedures included Geographic Position System coordinates per household compared to cluster location, household response rate, number of visits, duration of data collection per child measured, duration of data collection per acutely malnourished child, the age distribution of measured children, terminal digit rounding, and distribution dispersion of MUAC values. Data reviews were scheduled in between sequenced surveys.

### 2.4 Calculation of coverage estimates from secondary sources

We derived indirect coverage according to available administrative data. This method has been described elsewhere and involves dividing reported admissions by the expected children with SAM according to the estimated population 6–59 months multiplied by the SAM prevalence by an incidence correction factor (16). Source and methods per country are detailed in Supplementary File 2 (26–38).

### 2.5 Definitions

Severe acute malnutrition was defined as MUAC <115 mm and/or edema, whereas MAM was defined as MUAC <125 mm and MUAC > = 115 mm without edema. Point coverage was calculated as the number of cases currently in treatment divided by the number of cases currently eligible for treatment. Program enrolment was evaluated based on a review of treatment cards, the presence of therapeutic food sachet, and/or parent recall. Cases meeting the national definition of a defaulter -- two consecutive weeks of missed visits for SAM (3 weeks in Burkina Faso), and 4 weeks of missed visits for MAM – were not considered as currently in treatment.

### 2.6 Statistical analysis

Analyses were conducted using R (V.4.0.4) (39). The *gtsummary* package was used for summarizing descriptive statistics and differences in coverage (40). Kruskal-Wallis rank-sum tests for continuous variables and Chi-squared test of independence for categorical variables with all expected cell counts > = 5 were used (40). Spearman correlation coefficients were used to test the association between measured and expected coverage and measured and expected prevalence (40). Survey weights for coverage estimates accounted for any unequal selection probabilities based on the sample design, as well as varying response rates in different clusters.

## 3 Results

The final samples ranged from 1,877 households in Baro, Chad to 15,026 households in Nyemba, DRC (Table 2). The household response rate was higher than 90% in most surveys, except for two in Niger. Absentee rates ranged from <0.1% in Sig-Noghin, Burkina Faso, to 17% in Filingué, Niger. Refusal rates ranged from 0 in Mangalmé, Chad, to 8.6% in Bogodogo, Burkina Faso. The surveys recruited 10% or more of the targeted catchment population in 7 of 11 surveys.

**Table 2:** Survey completion (households).

	Burkina Faso			Chad			DRC		Niger		
	Bogodogo, *N* = 7,153	Boulmiougou, *N* = 7,857	Sig-Noghin, *N* = 7,681	Baro, *N* = 1,877	Mangalmé, *N* = 3,479	Melfi, *N* = 3,156	Kalemie, *N* = 6,472	Nyemba, *N* = 15,026	Balleyara, *N* = 5,336	Filingué, *N* = 4,684	Oullam, *N* = 4,639
Visit result											
Completed	6,463 (90%)	7,690 (98%)	7,619 (99%)	1,802 (96%)	3,320 (95%)	3,135 (99%)	6,320 (98%)	14,791 (98%)	4,753 (89%)	3,904 (83%)	4,413 (95%)
Absent	73 (1.0%)	12 (0.2%)	7 (<0.1%)	17 (0.9%)	155 (4.5%)	11 (0.3%)	54 (0.8%)	52 (0.3%)	576 (11%)	779 (17%)	224 (4.8%)
Absent- no second visit^1^	0 (0%)	0 (0%)	0 (0%)	54 (2.9%)	0 (0%)	4 (0.1%)	21 (0.3%)	64 (0.4%)	1 (<0.1%)	0 (0%)	1 (<0.1%)
Partially completed^2^	2 (<0.1%)	3 (<0.1%)	1 (<0.1%)	2 (0.1%)	4 (0.1%)	1 (<0.1%)	4 (<0.1%)	6 (<0.1%)	0 (0%)	0 (0%)	0 (0%)
Refused	615 (8.6%)	152 (1.9%)	54 (0.7%)	2 (0.1%)	0 (0%)	5 (0.2%)	73 (1.1%)	113 (0.8%)	6 (0.1%)	1 (<0.1%)	1 (<0.1%)
% of the catchment population represented by the survey	0.4%	3.8%	9.8%	10.3%	11.7%	14.7%	11.4%	79.7%	23.3%	6.3%	10.0%

^1^These households did not follow protocol, which was a second visit for all absent households.

^2^Data for partially completed questionnaires used in the analysis.

Demographic characteristics, nutritional status, and access to care of the final sample are presented in Table 3. The sex distribution of measured children was nearly equal across contexts (49–51% male). The mean age of all measured children ranged from 31–32 months. Proximity to the health facility varied between contexts. Whereas most measured children lived within 1 hour of the health facility in Burkina Faso, DRC, and Niger, less than 50% of children lived within 1 hour of the health facility in Chad. Significant differences in distance to the nearest facility were detected between catchment areas in all four countries.

**Table 3:** Characteristics of children surveyed.

	Burkina Faso			Chad			DRC		Niger		
	Bogodogo, *N* = 2,639	Boulmiougou, *N* = 2,675	Sig-Noghin, *N* = 2,756	Baro, *N* = 2,087	Mangalmé, *N* = 2,750	Melfi, *N* = 3,211	Kalemie, *N* = 4,775	Nyemba, *N* = 9,556	Balleyara, *N* = 3,504	Filingué, *N* = 2,806	Ouallam, *N* = 4,276
Demographic information											
Male, *n*%	1,323 (50%)	1,345 (50%)	1,387 (50%)	1,055 (51%)	1,352 (49%)	1,638 (51%)	2,459 (51%)	4,781 (50%)	1,793 (51%)	1,393 (50%)	2,120 (50%)
Age in months, mean (standard deviation)	31 (14)	31 (14)	32 (15)	32 (16)	32 (15)	31 (16)^***^	31 (15)	32 (15)^**^	31 (15)	31 (15)	32 (15)^*^
Distance to nearest treatment site <1 h, *n* (%)	1,800 (68%)	2,267 (85%)	2,501 (91%)^***^	966 (47%)	1,117 (42%)	1,470 (46%)^***^	4,169 (87%)	8,898 (93%)^***^	2,195 (64%)	1,362 (50%)	2,525 (61%)^***^
Anthropometric results											
MUAC in mm, mean (standard deviation)	148 (13)	147 (12)	146 (12)^***^	141 (13)	140 (12)	139 (12)^***^	145 (13)	145 (12)	142 (12)	141 (12)	143 (12)^***^
GAM (95% CI)^1^	2.2% (1.5–2.9%)	2.6% (1.7–3.4%)	2.6% (1.7–3.5%)	7.5% (4.8–10.1%)	6.0% (3.8–8.2%)	8.5% (6.4–10.5%)	7.3% (5.5–9.0%)	4.3% (3.8–4.8%)	5.5% (4.7–6.4%)	6.3% (5.0–7.6%)	7.0% (4.5–9.5%)
SAM (95% CI)^2^	0.6% (0.3–1.0%)	1.2% (0.7–1.8%)	1.2% (0.6–1.7%)	2.5% (1.3–3.6%)	1.6% (0.7–2.4%)	1.8% (1.0–2.7%)	2.7% (1.7–3.6%)	1.3% (1.1–1.4%)	1.5% (1.1–1.9%)	1.6% (1.0–2.3%)	2.1% (0.8–3.4%)
Edema (*n*)	0	0	0	3	0	1	7	6	2	0	1

*p < 0.05; **p < 0.01; ***p < 0.001.

Confidence intervals are weighted for survey design.

^1^MUAC < 125 mm and/or edema.

^2^MUAC < 115 mm and/or edema.

GAM, Global Acute Malnutrition; MUAC, mid-upper arm circumference.

Prevalence of Global Acute Malnutrition (GAM), according to MUAC and edema was highest in Chad (6.0–8.5%), followed by Niger (5.5–7.0%), DRC (4.3–7.3%), and Burkina Faso (2.2–2.6%). Prevalence of Severe Acute Malnutrition (SAM) according to MUAC and edema ranged from 1.3–2.7% in DRC, 1.6–2.5% in Chad, 1.5–2.5% in Niger, and 0.6–1.2% in Burkina Faso. The highest number of edema cases were identified in DRC, followed by Chad and Niger. No edema cases were identified in Burkina Faso.

Point coverage for SAM ranged from nearly 2.2% in Filingué, Niger, to 45.1% in Baro, Chad (Table 4). Fewer than 1 in 5 eligible children were enrolled in treatment in 9 of 11 catchment areas. MAM coverage was less than 10% in all assessed catchment areas.

**Table 4:** Coverage of malnutrition treatment among acutely malnourished children aged 6–59 months.

	Burkina Faso			Chad			DRC		Niger		
	Bogodogo, *n* = 24	Boulmiougou, *n* = 40	Sig-Noghin, *n* = 32	Baro, *n* = 48	Mangalmé, *n* = 55	Melfi, *n* = 58	Kalemie, *N* = 127	Nyemba, *N* = 122	Balleyara, *N* = 53	Filingu*é, N* = 44	Ouallam, *N* = 90
Point coverage (SAM)	13.0% (0–28.4%)	5.3% (0–13.4%)	13.0% (1.6–24.4%)	45.1% (25.0–65.3%)	34.5% (19.2–49.8%)	13.2% (1.5–24.8%)	6.4% (0.0–13.8%)	9.0% (4.2–13.8%)	13.5% (2.9–24.0%)	2.2% (0.0–6.9%)	15.4% (4.9–25.9%)
Expected coverage (SAM)	5%	5%	5%	30%	35%	28%	25%	30%	20%	25%	25%
							*N* = 219	*N* = 291	*N* = 140	*N* = 128	*N* = 203
Point coverage (MAM)							1.4% (0.0–3.0%)	0.3% (0–0.6%)	2.1% (0–4.3%)	2.9% (0–7.3%)	6.8% (3.3–10.4%)

Confidence intervals are weighted for survey design.

MAM, Moderate Acute Malnutrition; SAM, Severe Acute Malnutrition.

**Figure 1:**
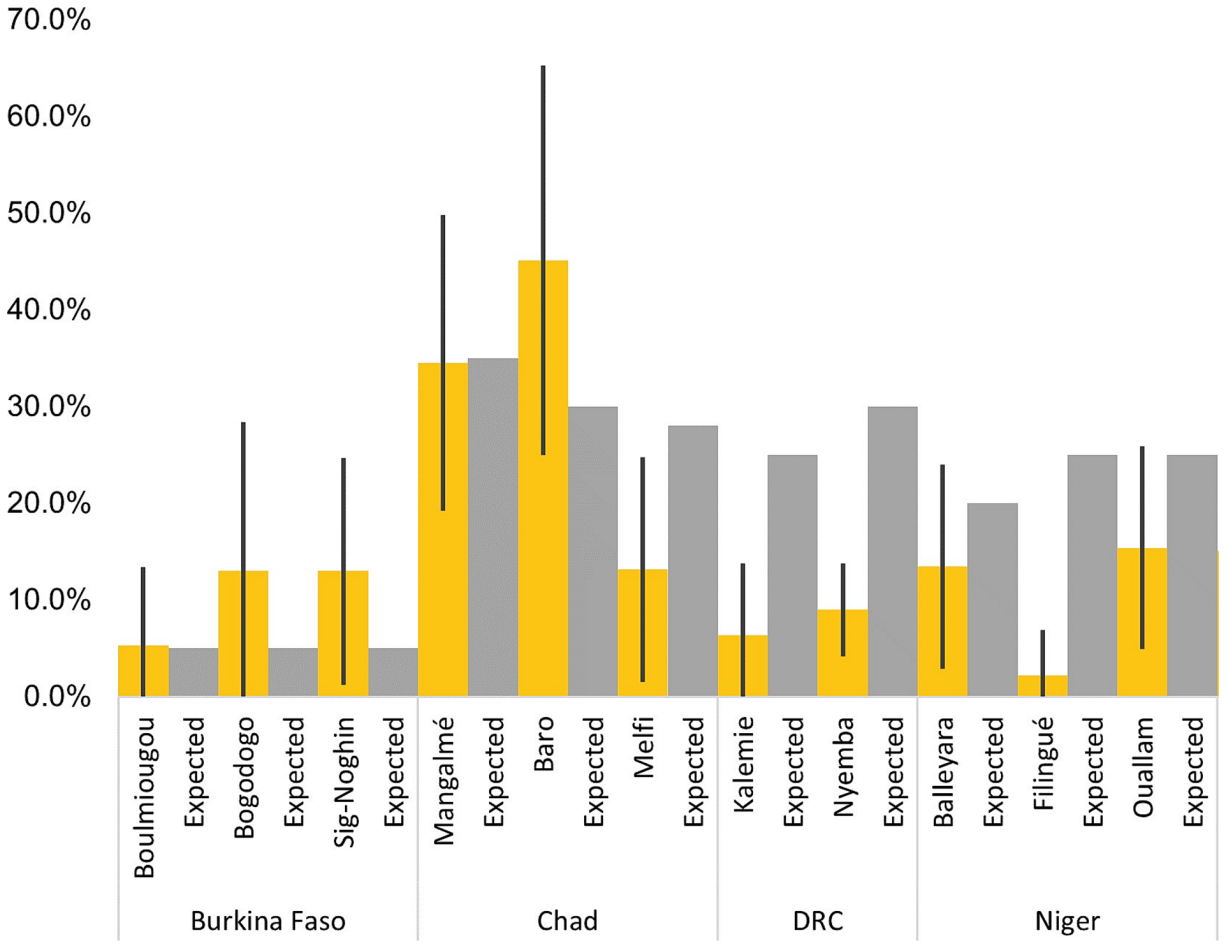
Measured coverage vs. practitioner expectations.

Measured SAM coverage did not correlate either with measured SAM prevalence (Rho 0.35; *p*-value 0.30) or with expected coverage estimated *a priori* (Rho 0.45; *p*-value 0.16). In 7 of 11 assessed administrative areas, the confidence interval of the measured coverage included the expected coverage estimate. In the other administrative areas with available, expected coverage, the upper confidence limit of the measured coverage was lower than the expected coverage (Figure 1).

Administrative estimates of coverage were available in Burkina Faso, Chad, and Niger (Supplementary Tables 2.1.1–2.1.3). In Burkina Faso, administrative estimates from 2020 range from 14.3–23.7%, but exceeded 100% in 2021 (Supplementary Table 2.1.1). In Niger, administrative estimates from 2020 data also exceed 100%. In Chad, we obtained monthly admission data from 2019 and 2020. Monthly coverage derived from administrative data varied from thirty to sixty-eight percentage points in the same year (Supplementary Table 2.1.2). In 2020, the median monthly coverage ranged from 22% in Mangalmé to 62.6% in Baro. In 2019, the median monthly coverage ranged from 39.1% in Mangalmé to 67.4% in Baro.

Evaluated factors associated with coverage of SAM and MAM are presented in Tables 5, 6, respectively. With respect to child characteristics, coverage did not differ significantly by sex or age of the child, but did vary by MUAC at assessment. In DRC and Niger, mean MUAC of covered children with SAM was significantly lower than the mean MUAC of non-covered children. Prior training of the caregiver in identification and referral of malnutrition, as evaluated by enrollment in the ‘Family MUAC’ program was also associated with coverage. In Chad, 18% of covered SAM children had one or more family members who had been previously trained in the Family MUAC approach, compared to 3.6% of non-covered SAM children. Geographic access was also a significant predictor of coverage in select countries. In Chad, a significantly higher percentage of covered children lived within an hour of the treatment facility, compared to non-covered children.

**Table 5:** Characteristics of covered and non-covered SAM children.

	Burkina Faso		Chad		DRC		Niger	
	Covered, *N* = 13	Non-covered, *N* = 83	Covered, *N* = 49	Non-covered, *N* = 112	Covered, *N* = 19	Non-covered, *N* = 230	Covered, *N* = 22	Non-covered, *N* = 165
Male, *n* (%)	6 (46%)	29 (35%)	16 (33%)	47 (42%)	5 (26%)	112 (49%)	11 (50%)	62 (38%)
Age in months, mean (SD)	16 (12)	17 (10)	15 (7)	16 (11)	15 (7)	20 (13)	13 (6)	17 (11)
Child MUAC (mm), mean (SD)	112 (2)	113 (3)	110 (6)	110 (7)	108 (7)	112 (7)***	109 (5)	110 (6)**
Previously trained in Family MUAC, *n* (%)			9 (18%)	4 (3.6%)**	0 (0%)	1 (0.4%)	5 (23%)	35 (21%)
Distance to nearest treatment site less than 1 h, *n* (%)	11 (85%)	68 (82%)	27 (55%)	25 (23%)***	15 (79%)	192 (83%)	13 (59%)	109 (67%)

*p < 0.05; **p < 0.01; ***p < 0.001.

MUAC, mid-upper arm circumference.

**Table 6:** Characteristics of covered and non-covered MAM children.

	DRC		Niger	
	Covered, *N* = 4	Non-covered, *N* = 506	Covered, *N* = 21	Non-covered, *N* = 450
Male, *n* (%)	3 (75%)	224 (44%)	5 (24%)	193 (43%)
Age in months, mean (SD)	18 (9)	19 (11)	16 (7)	19 (11)
Child MUAC (mm), mean (SD)	122 (3)	121 (3)	120 (3)	120 (3)
Previously trained in Family MUAC, *n* (%)	0 (0%)	3 (0.6%)	4 (19%)	90 (20%)
Distance to nearest treatment site less than 1 h, *n* (%)	2 (50%)	438 (87%)	14 (67%)	233 (53%)***

***p < 0.001.

MUAC, mid-upper arm circumference.

## 4 Discussion

This analysis presents treatment coverage for acute malnutrition using representative cluster-based population survey methods in eleven administrative areas of four high-burden countries. Coverage of treatment for acute malnutrition was universally below global standards (7) but variable across and within countries. The point estimates of coverage suggest that fewer than one in five eligible SAM children were enrolled in treatment in nine administrative areas. These results were obtained in the areas where treatment was available prior to the survey. In Chad, one administrative area had significantly lower SAM coverage than the other areas assessed in the same country.

Given low coverage, we aimed to characterize individual, household, and population level characteristics associated with enrollment in treatment. Little evidence exists on child-level factors that may be correlated with coverage. Our findings suggest no significant differences in SAM or MAM coverage by child age in the populations assessed. We also did not detect sex differences in SAM or MAM coverage, despite evidence to suggest sex differences in prevalence (41, 42).We did identify differences in MUAC across covered and non-covered populations in two countries. In DRC and Niger, enrolled cases had lower MUAC than non-enrolled cases. As the time to presentation is a key determinant of successful treatment in the CMAM model, this is an interesting finding that should be monitored with routine program data (43). It is possible that in some contexts, more visible signs of malnourishment (i.e., lower MUAC) may increase the urgency to take the child to be screened or stay enrolled in the program.

At the household level, we found a significant association between individual treatment status and distance to the health facility for SAM treatment coverage in Chad. Here, the relationship was as expected – significantly higher percentage of covered SAM cases (Chad) lived within the 1 h traveling distance to the treatment facility compared to non-covered cases. In all other assessed countries, this relationship was non-significant. Despite evidence that improving geographic access to health facilities increases the use of maternal and child health services (44, 45), and distance to the facility is a well-established primary barrier to CMAM coverage (46), little routine work is done to map locations to CMAM sites and understand the spatial distribution of coverage (17). Many CMAM programs track treatment availability (the number of facilities offering services) and effectiveness (cure rates), without understanding accessibility (the population who can reasonably use the service) (47). Targets ensuring geographic access to care are rarely, if ever, set.

An association between individual treatment status and caregiver involvement in screening was observed in one country (Chad). To improve early detection and referral of acutely malnourished children, the Family MUAC approach trains caregivers to screen children at home on a regular basis, as opposed to waiting for screening by a community-based volunteer or at the facility. In Chad, a significantly higher percentage of covered children had a caregiver who was previously trained in the Family MUAC approach, compared to non-covered children. While the cross-sectional and observational nature of our study prevents us from making causal inferences, this finding is in line with promising but limited peer-reviewed evidence and operational findings on the approach’s effectiveness (48, 49).

At the population level, SAM treatment coverage was unrelated to SAM prevalence. Despite their effectiveness, CMAM programs are regularly underfunded and not integrated into the national healthcare systems. Where resources are limited, we would assume areas with higher prevalence might be prioritized for services (50). However, this was not observed in our study, or it may be that there is a disconnect in supply through treatment provision and demand detected in enrollment and retention in the program. Overall, the factors that drive wasting may also be associated with lower coverage, which was supported by our study. While we are unable to draw determinations from our surveys regarding population density to facility, we note in Niger estimated ratio of children 6 to 59 months per facility ranged widely between the three districts, and that coverage was lowest where this ratio was nearly two-fold higher than the other districts. In Baro, Chad, where population density was the lowest, we note the highest SAM coverage (45.1%). While we do not note a consistent trend in estimated population density to coverage, we feel that our work supports efforts to further decentralize care to CHW’s such that catchment areas are feasible geographic and population size (4).

Because we used novel methods to assess coverage, we compared our findings to previous estimates derived using conventional methods and secondary data sources. We first compared our findings to estimates derived using administrative data (Supplementary File 2). Our data is cross-sectional, directly measuring the percentage of eligible children enrolled at one point in time, whereas estimates derived from programmatic data reflect enrollment over a given period (i.e., monthly or annually). In Burkina Faso, administrative estimates from 2020 were somewhat similar to our findings (Supplementary Table 2.1.1). This was the exception, as administrative coverage estimates from 2021 in Burkina Faso and in other countries ranged from much lower to much higher than our findings, sometimes implausibly exceeding 100%. This may be due to outdated population and/or prevalence estimates. For example, in Niger, coverage estimates were clearly implausible (ranging from 188 to 235%) (Supplementary Table 2.1.3). It is important to note that in this context, prevalence figures for combined SAM (all anthropometric criteria) were not available- only those disaggregated as SAM by MUAC/ edema, and SAM by WHZ. While this likely contributed to an underestimation of expected SAM admissions, the direction and magnitude of the discrepancy varied by country and year.

In Chad, we obtained monthly admission data from 2019 and 2020. Acute malnutrition prevalence is driven by seasonality, climate, and conflict crises, and if enrollment does not increase during the peaks, coverage will decrease when the burden surges (51). Our findings in Chad suggest the population estimates for prevalence or population are not representative of some months of the year, and/or coverage fluctuated by month. Monthly coverage derived from administrative data varied from thirty to sixty-eight percentage points in the same year (Supplementary Table 2.1.2). In 2020, the median monthly coverage was higher than our findings in two districts and lower in one district. In 2019, the median monthly coverage was higher than our findings in all three districts.

As administrative coverage estimates leverage routinely collected data, they also rely on the accuracy and precision of this data (16). We use the general incidence correction factor 1.6, whereas recent work suggests this may be an underestimate in many contexts (52–54).Ultimately, our findings suggest that coverage estimates derived using administrative data are unreliable. The direct measurements from our data help assess the accuracy of coverage estimates from administrative data where they are available.

We also compared our findings to SQUEAC and SLEAC, the two most frequently used methods for direct assessment of acute malnutrition coverage. Both are based on data collected through active and adaptive case finding, which is then adjusted by a Bayesian model that relies on numerous assumptions and estimated parameters. SQUEAC produces coverage estimates with an associated 95% confidence interval, whereas SLEAC classifies treatment coverage as low, medium, or high based on contextualized thresholds.

Coverage estimates produced by SQUEAC or SLEAC were only available for Chad and Niger and were quite outdated (from 2013 to 2016) (Supplementary File 2, Supplementary Tables 2.2.1, 2.2.2). These prior estimates from SQUEAC and SLEAC (except for one in Mangalme, Chad, in 2015) were considerably higher than the coverage estimates obtained in our study. While it is possible that coverage has decreased over time in some areas, that seems an unlikely explanation for the differences, given active work in all countries to scale up coverage (12). Our findings support the theoretical concern that the active and adaptive case finding at the core of the SQUEAC method carries a risk of upward bias (55). Our findings suggest a similar risk with SLEAC, though the broad classification thresholds make a comparison to our point coverage estimates difficult. In one case in Chad, SLEAC coverage classification was higher than our findings, whereas in the other, it was lower (2015). In Niger, the SLEAC coverage classification was higher than our findings in all cases (2014 and 2015).

Lastly, we compared our findings to practitioner expectations (Supplementary File 2, Supplementary Table 2.3). Expected coverage estimates were estimated by IRC technical staff in each country office, informed by stakeholder consultation, and a review of factors that can boost or inhibit coverage, including the food security situation, partner presence prior to the project, availability of MAM treatment, insecurity/ physical accessibility, population displacement, and available coverage survey data. The specific approach to this exercise varied by office, but in all cases, it was not as extensive as the formulation of a prior per the SQUEAC and SLEAC methodologies (17). In 7 contexts, predicted coverage for SAM was within the 95% CI for measured coverage. In 4 other contexts, predicted coverage for SAM was higher than the 95% CI for measured coverage, indicating practitioners were overly optimistic regarding coverage.

In addition to presenting findings with respect to coverage, we demonstrated the feasibility of population-based methods themselves in a variety of contexts. In contexts where SAM prevalence was higher, data collection required 5–7 days of fieldwork. In lower prevalence contexts, 14–20 days were required. We made a reasoned choice to select clusters using spatial stratified systematic sampling or PPS depending on the context. Spatial stratified systematic sampling did not require weighting analyses for unequal probability of cluster selection, and thus we recommend practitioners use this method.

The key strength of this study is the direct measurement of acute malnutrition treatment coverage using gold-standard population-based methods. This approach avoids the imprecision and bias potentially introduced by pre-assessment assumptions, post-hoc modeling, or correction factors. These methods resulted in direct population representative estimates for administrative areas where coverage estimates were previously unavailable, outdated, and/or produced by methods with critical risks of bias. The exhaustive sampling of children within selected clusters allowed us to examine individual and household-level factors associated with coverage. We present novel findings on the association of prevalence and coverage using the same data collection method, and we also make comparisons to existing coverage estimates.

Our study has several limitations. First, our survey’s case-identification criteria, while aligned with the community-based MUAC and edema screening protocol in all four countries, did not account for children malnourished by weight-for-height z-score without MUAC deficiency and/or edema. Mid-upper arm circumference, weight-for-height z-scores, and edema identify overlapping but not identical populations, which varies by setting (56). All anthropometric criteria aim to identify the children most at risk of death due to undernutrition. MUAC cut-offs have been shown to effectively identify children at risk of death (57), but MUAC identifies a younger and more female treatment population than WHZ (57–59). Based on programmatic data from the implementing areas, 10.4 to 55.1% of all children admitted for treatment presented with low weight-for-height alone, and therefore, would not have been included in the denominator of our coverage assessments. It is likely that the coverage for SAM children malnourished by weight-for-height z-score and not MUAC and/or edema is even lower than identified in our surveys and community referrals using MUAC and edema criterion, as these children are only detected as malnourished through passive screening at the health facility. Socio-demographic associations with coverage should also be interpreted with caution, as they may differ for children who are malnourished by weight-for-height z-score and not MUAC and/or edema.

Second, we measured a few covariates at the individual, household, and population level to explore in relation to coverage. Additional socio-demographic factors which may be associated with coverage, such as socioeconomic status, health indicators, and infant and young child feeding practices, were not included in the data collection tool. At the population level, we did not systematically collect information on programs in the area operated by other partners, to assess the relationships between programmatic interventions and coverage. Contextual information was, at times difficult to generalize across entire administrative areas, especially regarding screening regularity and supply availability, which can vary across facilities. Our surveys were conducted during distinct seasons not necessarily aligned with malnutrition peaks; future work would benefit from investigating seasonal differences in coverage. Unlike SQUEAC’s, our surveys did not integrate qualitative assessment to explain potential drivers of low or high coverage.

Finally, we conducted additional coverage surveys the catchment areas of facilities supported by the project Somalia. Due to the difficulty of obtaining a sampling framework representative of the catchment areas in this context, we exclude results from this paper but reported them for programming purposes.

## 5 Conclusion

Improving treatment coverage is critical for improving a program’s cost efficiency in any given context (16, 45, 60). Our findings suggest that, in four high-burden countries, many more children are eligible for treatment than are enrolled. In the global acute malnutrition treatment landscape, this calls for increased efforts to improve treatment coverage through decentralized delivery, simplified approaches, and scaled up screening efforts. The methods we present provide important context to coverage estimates derived from administrative data and factor in fewer assumptions than existing discrete methods to measure coverage. Strengthening both routine program data and direct coverage assessment methodologies can help understand met needs and changes over time, ultimately improving the scale and effectiveness of SAM programs. Future work should consider the opportunity to strengthen routine monitoring systems such that administrative estimates of coverage can be reliably used in triangulation with results from direct population-representative surveys, such as the methods we describe. Future surveys using this methodology should investigate the feasibility of incorporate weight and height measurements such that coverage can be calculated by respective admission criteria of SAM and MAM.

## Data Availability

The raw data supporting the conclusions of this article will be made available by the authors, without undue reservation.
