# Supplementary material for: Estimating program coverage in the treatment of acute malnutrition using population-based cluster survey methods: results from surveys in Burkina Faso, Chad, Democratic Republic of the Congo, and Niger
Source: Front Public Health. 2025 Mar 25;13:1513567. doi: 10.3389/fpubh.2025.1513567 (PMC11975588; doi:10.3389/fpubh.2025.1513567)
Supplement: Supplementary file 1 [file Supplementary_file_1.docx]

|  | **Burkina Faso** | | | **Chad** | | | **DRC** | | **Niger** | | |
| --- | --- | --- | --- | --- | --- | --- | --- | --- | --- | --- | --- |
|  | Health district | | | Health district | | | CMAM Avancé catchment areas within health zones | | Health district | | |
|  | Bogodogo | Boulmiougou | Sig-Noghin | Baro | Mangalm é | Melfi | Kalemie | Nyemba | Balleyara | Filingué | Ouallam |
| **Sampling parameters** | | | | | | | | | | | |
| Expected coverage (SAM) | 5% | 5% | 5% | 30% | 35% | 28% | 25% | 30% | 20% | 25% | 25% |
| Expected non-response | 5% | 5% | 5% | 5% | 5% | 5% | 5% | 5% | 5% | 5% | 5% |
| Precision | 7.0% | 6.5% | 7.0% | 12% | 12% | 12% | 10% | 13% | 10% | 11% | 12% |
| Prevalence of SAM by MUAC <115 mm and/or edema | 0.4% | 0.4% | 0.4% | 1.9% | 1.9% | 1.9% | 1.3% | 1.3% | 1.0% | 1.0% | 1.0% |
| Percentage of the population 6-59 months | 12.7% | 12.7% | 12.7% | 22.0% | 22.0% | 22.0% | 17.9% | 17.9% | 16.6% | 16.6% | 16.6% |
| Average cluster size (total population) | 2043 | 2263 | 2032 | 869 | 595 | 285 | 1124 | 530 | 924 | 1081 | 758 |
| **Calculated sample size** | | | | | | | | | | | |
| SAM children | 37 | 43 | 37 | 56 | 61 | 54 | 72 | 48 | 61 | 60 | 50 |
| Clusters | 38 | 40 | 38 | 18 | 26 | 48 | 31 | 43 | 42 | 35 | 42 |

**Supplementary Table 1. Sample size parameters**
