# Supplementary material for: Estimating program coverage in the treatment of acute malnutrition using population-based cluster survey methods: results from surveys in Burkina Faso, Chad, Democratic Republic of the Congo, and Niger
Source: Front Public Health. 2025 Mar 25;13:1513567. doi: 10.3389/fpubh.2025.1513567 (PMC11975588; doi:10.3389/fpubh.2025.1513567)
Supplement: Supplementary file 2 [file Supplementary_file_2.docx]

**Supplementary File 2: Coverage Estimates from Secondary Sources**

**Supplementary Table 2.1. Coverage Survey Estimates using Administrative Data**

**Supplementary Table 2.1.1 Burkina Faso**

|  | **Direct coverage estimates  (Our Data)** | **Administrative Coverage Estimates** | | |
| --- | --- | --- | --- | --- |
| **Administrative Area** |  | **Coverage** | **Source** | **Methods** |
| Bogodogo | 13.0%  (0-28.4%) | 155.5% | 26. Ministere de la Sante Burkina Faso ; Direction Générale des études et des statistiques sectorielles. Annuaire statistique 2021. | Administrative coverage calculated as follows:  Numerator is reported annual discharges per health district  Denominator is expected SAM children per health district |
| Boulmiougou | 5.3%  (0-13.4%) | 112.3% |  |  |
| Sig-Noghin | 13.0%  (1.6-24.4%) | 119.9% |  |  |
| Bogodogo | 13.0%  (0-28.4%) | 13.6% | 27. Ministere de la Sante Burkina Faso ; Direction Générale des études et des statistiques sectorielles. Annuaire statistique 2020. |  |
| Boulmiougou | 5.3%  (0-13.4%) | 14.3% |  |  |
| Sig-Noghin | 13.0%  (1.6-24.4%) | 23.7% |  |  |

**Supplementary Table 2.1.2 Chad**

| **Administrative Area** | **Direct coverage estimates  (Our Data)** | **Administrative Coverage Estimates** | | | |
| --- | --- | --- | --- | --- | --- |
|  |  | **Coverage** | **Month** | **Source** | **Methods** |
| Baro | 45.1%  (25.0-65.3%) | 53.4% | January 2020 | Reported new SAM admissions by district. Ministry of Health. 2020.  28. 2019 National SMART Survey : Guera province.  Population figures. Ministry of Health. 2019. | Administrative coverage calculated as follows:  Numerator is reported monthly new admissions^[[1]](#footnote-1)^  Denominator is expected SAM children based on:  1) 2019 expected population 6-59 months, Ministry of Health^[[2]](#footnote-2)^*  2) Prevalence SAM, National SMART^[[3]](#footnote-3)^ *  3) Incidence correction factor (1.6)^[[4]](#footnote-4)^ |
|  |  | 48.0% | February 2020 |  |  |
|  |  | 96.0% | March 2020 |  |  |
|  |  | 52.9% | April 2020 |  |  |
|  |  | 57.7% | May 2020 |  |  |
|  |  | 84.8% | June 2020 |  |  |
|  |  | 72.4% | July 2020 |  |  |
|  |  | 71.6% | August 2020 |  |  |
|  |  | 92.6% | September 2020 |  |  |
|  |  | 63.8% | October 2020 |  |  |
|  |  | 56.8% | November 2020 |  |  |
|  |  | 61.4% | December 2020 |  |  |
|  |  | **62.6% (48.0-96.0%)** | **2020 Median** **(Min, Max)** |  |  |
| Mangalmé | 34.5%  (19.2-49.8%) | 24.0% | January 2020 |  |  |
|  |  | 17.9% | February 2020 |  |  |
|  |  | 38.1% | March 2020 |  |  |
|  |  | 30.3% | April 2020 |  |  |
|  |  | 35.8% | May 2020 |  |  |
|  |  | 13.0% | June 2020 |  |  |
|  |  | 12.2% | July 2020 |  |  |
|  |  | 20.4% | August 2020 |  |  |
|  |  | 28.2% | September 2020 |  |  |
|  |  | 19.3% | October 2020 |  |  |
|  |  | 17.1% | November 2020 |  |  |
|  |  | 7.9% | December 2020 |  |  |
|  |  | **22.0% (7.9-38.1%)** | **2020 Median (Min, Max)** |  |  |
| Melfi | 13.2%  (1.5-24.8%) | 45.2% | January 2020 |  |  |
|  |  | 13.7% | February 2020 |  |  |
|  |  | 75.4% | March 2020 |  |  |
|  |  | 47.0% | April 2020 |  |  |
|  |  | 81.8% | May 2020 |  |  |
|  |  | 25.1% | June 2020 |  |  |
|  |  | 43.3% | July 2020 |  |  |
|  |  | 74.0% | August 2020 |  |  |
|  |  | 54.0% | September 2020 |  |  |
|  |  | 64.1% | October 2020 |  |  |
|  |  | 43.3% | November 2020 |  |  |
|  |  | 52.9% | December 2020 |  |  |
|  |  | **51.6% (13.7-81.8%)** | **2020 Median (Min, Max)** |  |  |
| Baro | 45.1%  (25.0-65.3%) | 60.9% | January 2019 | Reported new SAM admissions by district. Ministry of Health. 2019.  28. 2019 National SMART Survey : Guera province  29. Population figures. Ministry of Health. 2019. | Administrative coverage calculated as follows:  Numerator is reported monthly new admissions^[[5]](#footnote-5)^  Denominator is expected SAM children per health district based on:  1) 2019 expected population 6-59 months, Ministry of Health *  2) Prevalence SAM, National SMART^2^ *  3) Incidence correction factor (1.6)^4^ |
|  |  | 48.7% | February 2019 |  |  |
|  |  | 69.9% | March 2019 |  |  |
|  |  | 60.2% | April 2019 |  |  |
|  |  | 65.5% | May 2019 |  |  |
|  |  | 45.3% | June 2019 |  |  |
|  |  | 63.1% | July 2019 |  |  |
|  |  | 60.4% | August 2019 |  |  |
|  |  | 80.2% | September 2019 |  |  |
|  |  | 83.3% | October 2019 |  |  |
|  |  | 82.4% | November 2019 |  |  |
|  |  | 88.9% | December 2019 |  |  |
|  |  | **67.4% (45.3-88.9%)** | **2019 Median (Min, Max)** |  |  |
| Mangalmé | 34.5%  (19.2-49.8%) | 48.1% | January |  |  |
|  |  | 24.7% | February |  |  |
|  |  | 54.9% | March |  |  |
|  |  | 46.8% | April |  |  |
|  |  | 59.4% | May |  |  |
|  |  | 60.2% | June |  |  |
|  |  | 58.5% | July |  |  |
|  |  | 29.3% | August |  |  |
|  |  | 26.1% | September |  |  |
|  |  | 25.4% | October |  |  |
|  |  | 16.4% | November |  |  |
|  |  | 19.3% | December |  |  |
|  |  | **39.1% (16.4-60.2%)** | **2019 Median (Min, Max)** |  |  |
| Melfi | 13.2%  (1.5-24.8%) | 57.9% | January |  |  |
|  |  | 58.8% | February |  |  |
|  |  | 71.4% | March |  |  |
|  |  | 79.0% | April |  |  |
|  |  | 64.7% | May |  |  |
|  |  | 48.6% | June |  |  |
|  |  | 58.6% | July |  |  |
|  |  | 46.9% | August |  |  |
|  |  | 59.5% | September |  |  |
|  |  | 78.8% | October |  |  |
|  |  | 78.1% | November |  |  |
|  |  | 56.3% | December |  |  |
|  |  | **63.2% (46.9-79.0%)** | **2019 Median (Min, Max)** |  |  |

**Supplementary Table 2.1.3 Niger**

| **Administrative Area** | **Direct coverage estimates  (Our Data)** | **Administrative Coverage Estimates** | | |
| --- | --- | --- | --- | --- |
|  |  | **Coverage** | **Source** | **Methods** |
| Balleyara | 13.5%  (2.9-24.0%) | 235% | 30. Reported new admissions by district. DHIS2. 2020.  31. National Health Information Systems. 2020.  32. National SMART- Tillaberi province. 2020. | Indirect estimates of coverages using administrative data as follows:  Numerator is reported annual admissions per health district^[[6]](#footnote-6)^  Denominator is expected SAM children based on:  1) 2019 expected population 6-59 months, Ministry of Health *  2) Prevalence of SAM, National SMART^[[7]](#footnote-7)^ *  3) Incidence correction factor (1.6) |
| Filingué | 2.2%  (0.0-6.9%) | 218% |  |  |
| Ouallam | 15.4%  (4.9-25.9%) | 188% |  |  |

**Supplementary Table 2.2 Coverage Survey Estimates using SQUEAC and SLEAC methodologies**

**Supplementary Table 2.2.1 Chad**

| **Administrative Area** | **Direct coverage estimates  (Our Data)** | **Coverage** | **Source** | **Methods** |
| --- | --- | --- | --- | --- |
| Mangalmé | 34.5%  (19.2-49.8%) | Low (less than 20%) | 33. 2015 SLEAC survey | Direct classification of coverage using the SLEAC methodology. |
| Melfi | 13.2%  (1.5-24.8%) | Moderate (20-50%) |  |  |

**Supplementary Table 2.2.2 Niger**

| **Administrative Area** | **Direct coverage estimates  (Our Data)** | **Coverage** | **Source** | **Methods** |
| --- | --- | --- | --- | --- |
| Balleyara | 13.5%  (2.9-24.0%) | 57.5% (47.8% - 66.3%) | 34. 2016 SQUEAC survey | Direct classification of coverage using the SQUEAC methodology. |
| Filingué | 2.2%  (0.0-6.9%) | 43.9% (34.7% - 53.6%) |  |  |
| Ouallam | 15.4%  (4.9-25.9%) | 53.8% (43.6% - 63.5%) |  |  |
| Balleyara | 13.5%  (2.9-24.0%) | Moderate (20-35%) | 35. 2015 SLEAC survey | Direct classification of coverage using the SLEAC methodology. |
| Filingué | 2.2%  (0.0-6.9%) | Moderate (20-35%) |  |  |
| Ouallam | 15.4%  (4.9-25.9%) | Moderate (20-35%) |  |  |
| Balleyara | 13.5%  (2.9-24.0%) | Moderate (20-35%) | 36. 2014 SLEAC survey | Direct classification of coverage using the SLEAC methodology. |
| Filingué | 2.2%  (0.0-6.9%) | Moderate (20-35%) |  |  |
| Ouallam | 15.4%  (4.9-25.9%) | Moderate (20-35%) |  |  |
| Filingué | 2.2%  (0.0-6.9%) | 24.5% (14.2-38.9%) | 37. 2013 SQUEAC survey | Direct classification of coverage using the SLEAC methodology. |

**Supplementary Table 2.3. Practitioner Expectations**

**Supplementary Table 2.3.1 Burkina Faso**

| **Country** | **District** | **Direct coverage estimates  (Our Data)** | **Expected coverage, SAM- MUAC < 115 mm and/or edema** | **Information source** |
| --- | --- | --- | --- | --- |
| Burkina Faso | Boulmiougou | 5.3%  (0-13.4%) | 5% | 38. Enquête PCIMA IRC 2021 :  Rapid needs assessment in the Centre Region of Burkina Faso |
|  | Bogodogo | 13.0%  (0-28.4%) | 5% |  |
|  | Sig-Noghin | 13.0%  (1.6-24.4%) | 5% |  |

**Supplementary Table 2.3.2 Chad**

| **Country** | **District** | **Direct coverage estimates  (Our Data)** | **Expected coverage, SAM- MUAC < 115 mm and/or edema** | **Information source** |
| --- | --- | --- | --- | --- |
| Tchad | Mangalmé | 34.5%  (19.2-49.8%) | 35% | Review of previous coverage surveys, stakeholder consultation, review of programmatic data. |
|  | Baro | 45.1%  (25.0-65.3%) | 30% | Review of previous coverage surveys, stakeholder consultation, review of programmatic data. |
|  | Melfi | 13.2%  (1.5-24.8%) | 28% | Review of previous coverage surveys, stakeholder consultation, review of programmatic data. |

**Supplementary Table 3.3 RDC**

| **Country** | **District** | **Direct coverage estimates  (Our Data)** | **Expected coverage, SAM- MUAC**  **< 115 mm and/or edema** | **Information source** |
| --- | --- | --- | --- | --- |
| RDC | Kalemie | 6.4%  (0.0-13.8%) | 25% | Stakeholder consultation, review of programmatic data. |
|  | Nyemba | 9.0%  (4.2-13.8%) | 30% | Stakeholder consultation, review of programmatic data. |

**Supplementary Table 3.4 Niger**

| **Country** | **District** | **Direct coverage estimates  (Our Data)** | **Expected coverage, SAM- MUAC**  **< 115 mm and/or edema** | **Information source** |
| --- | --- | --- | --- | --- |
| Niger | Balleyara | 13.5%  (2.9-24.0%) | 20% | Review of previous coverage surveys, stakeholder consultation,  review of programmatic data. |
|  | Filingué | 2.2%  (0.0-6.9%) | 25% | Review of previous coverage surveys, stakeholder consultation,  review of programmatic data. |
|  | Ouallam | 15.4%  (4.9-25.9%) | 25% | Review of previous coverage surveys, stakeholder consultation,  review of programmatic data. |

1. Admissions in 2020 based on MUAC and/or edema admission criteria. [↑](#footnote-ref-1)
2. 2020 population figures not available. [↑](#footnote-ref-2)
3. Severe acute malnutrition by MUAC and/or edema. [↑](#footnote-ref-3)
4. Garenne M, Willie D, Maire B, et al.. Incidence and duration of severe wasting in two African populations. *Public Health Nutr* 2009;12:1974–82. [↑](#footnote-ref-4)
5. Admissions in 2020 based on MUAC and/or edema admission criteria. [↑](#footnote-ref-5)
6. Admissions in 2020 based on weight-for-height z-score, MUAC and/or edema admission criteria. [↑](#footnote-ref-6)
7. The prevalence for SAM is reported separately by weight for height and/or edema, and MUAC, in the 2020 National SMART report. Both indicate 0.7% as the SAM prevalence. The prevalence used in the denominator was 0.7%, but it is important to note that the combined SAM prevalence (child SAM by any admission criteria) is likely higher, thus resulting in an underestimate of expected SAM cases. [↑](#footnote-ref-7)
